# Genetics of a de novo origin of undifferentiated multicellularity

**DOI:** 10.1098/rsos.180912

**Published:** 2018-08-29

**Authors:** Matthew D. Herron, William C. Ratcliff, Jacob Boswell, Frank Rosenzweig

**Affiliations:** 1 Division of Biological Sciences, University of Montana, 32 Campus Drive, Missoula, MT 59801, USA; 2 School of Biological Sciences, Georgia Institute of Technology, 950 Atlantic Drive, Atlanta, GA 30332, USA

**Keywords:** *Chlamydomonas*, experimental evolution, genetics, major transitions, multicellularity

## Abstract

The evolution of multicellularity was a major transition in evolution and set the stage for unprecedented increases in complexity, especially in land plants and animals. Here, we explore the genetics underlying a de novo origin of multicellularity in a microbial evolution experiment carried out on the green alga *Chlamydomonas reinhardtii*. We show that large-scale changes in gene expression underlie the transition to a multicellular life cycle. Among these, changes to genes involved in cell cycle and reproductive processes were overrepresented, as were changes to *C. reinhardtii*-specific and volvocine-specific genes. These results suggest that the genetic basis for the experimental evolution of multicellularity in *C. reinhardtii* has both lineage-specific and shared features, and that the shared features have more in common with *C. reinhardtii*'s relatives among the volvocine algae than with other multicellular green algae or land plants.

## 1. Introduction

How and why organismal complexity increases are central questions in evolutionary biology. If we take the number of types of parts as an operational definition of complexity [1,2], it is clear that both the maximum and the average levels of complexity have increased from the origin of life to the present day (although the vast majority of life forms remain simple). Large increases in organismal complexity resulted from a series of events in which existing individuals combined to become components of a new kind of individual with parts specialized to play various roles. Such events are known as major transitions [3] or evolutionary transitions in individuality [4–6] and include the emergence of cellular life from groups of interacting molecular replicators, of eukaryotes from two prokaryotes, of multicellular organisms from unicells and of eusocial ‘superorganisms’ from individual animals. Among such transitions, the evolution of multicellular organisms from single-celled ancestors, and the further evolution of cellular differentiation, set the stage for unprecedented increases in complexity, especially in land plants and animals.

Multicellularity has evolved independently in at least 25 separate lineages, including the Eubacteria, Archaea and several lineages spanning the deepest divergences within the eukaryotes [7,8]. These independent origins of multicellularity are replicate natural experiments with the potential to reveal general principles involved in this transition. The predominant approach to studying these origins has been retrospective, using comparative methods. This approach has been productive in many cases, but it does suffer from important limitations. First, for most lineages, evidence of the earliest steps in the transition has been decimated by extinction and obscured by the limitations of the fossil record. Second, most such comparisons are with extant unicellular relatives, which are only approximate stand-ins for unicellular relatives; after all, they have been evolving just as long since the initial divergence as have the multicellular groups with which we are comparing them.

One way around these limitations is to use experimental microbial evolution to generate a de novo origin of multicellularity. Simple multicellular structures have evolved in response to predation in experiments using the green algae *Chlorella vulgaris* [9] and *Chlamydomonas reinhardtii* [10]. However, virtually nothing is known about the genetic changes underlying these evolutionary responses. In the yeast *Saccharomyces cerevisiae*, multicellular ‘snowflakes’ evolved in response to selection for an increased rate of settling out of liquid suspension [11]. These experiments show that simple multicellularity can evolve relatively quickly (approx. 100 to approx. 300 generations), given a strong selective pressure for increased size [9,12].

We have subjected the unicellular chlorophyte alga *C. reinhardtii* to selection for increased settling speed, resulting in the evolution of a simple multicellular life cycle [12]. The evolved isolate is characterized by unicellular propagules, which develop clonally into multicellular clusters. Clusters inoculated into fresh medium exhibit little movement or cell division. Around 4 h post-inoculation, motile unicells begin to disperse away from the parent cluster. By 8 h post-inoculation, many of these single-celled propagules have stopped swimming and begun to develop into multicellular clusters, which continue to grow by cell division over the remainder of the culture cycle.

*Chlamydomonas reinhardtii* is closely related to the colonial or multicellular volvocine algae in the families Tetrabaenaceae [13], Goniaceae [14] and Volvocaceae [15], which collectively form a multicellular clade. Because *C. reinhardtii* is nested within an otherwise unicellular clade, it is inferred to be primitively unicellular, i.e. to have no multicellular ancestors. The multicellular structures that evolved in our experiments thus represent a novel origin of simple multicellularity, not a reversion to an ancestral condition.

The life history of experimentally evolved *C. reinhardtii* is stable over successive generations in the absence of selection, indicating that it is heritable. Here, we explore the hereditary basis of this transition from a unicellular to a multicellular life cycle. Using a combination of whole-genome sequencing, bulked segregant analysis and genome-wide transcriptional analysis, we identify changes in gene structure and expression that distinguish an evolved multicellular clone from its unicellular ancestor.

## 2. Methods

### 2.1. Whole-genome sequencing

In the experiment described in Ratcliff *et al*. [12], outcrossed experimental populations of *C. reinhardtii* were subjected to selection for increased settling rate via low-speed centrifugation, resulting in the evolution of multicelled clusters in one of ten experimental populations. Multicellular isolate WRC01 (Will Ratcliff Clone 01) was derived from this population. Ten clones were initially isolated from the same experimental population, but Sanger sequencing of five unlinked loci revealed no genetic variation. We therefore treat WRC01 as representative of the algae from this population.

Populations in the evolution experiment reported on by Ratcliff *et al*. [12] were founded using a large pool of F2 progeny created by crossing 15 genetically diverse *C. reinhardtii* strains from the *Chlamydomonas* Resource Center (CC-124, CC-125, CC-1690, CC-1691, CC-2290, CC-2342, CC-2343, CC-2344, CC-2931, CC-2932, CC-2935, CC-2936, CC-2937, CC-2938 and CC-4414). The genomes of most of these parental strains had been previously sequenced to low coverage (approx. 10-fold average genomic coverage) using paired-end libraries on an Illumina platform [16]. To supplement these data, we generated our own paired-end sequencing data for four additional parental strains (CC-124, CC-1690, CC-1691 and CC-4414) and WRC01 (NCBI Sequence Read Archive accession numbers SAMN09580649 and SAMN09580651–4).

Algae for whole-genome sequencing were grown in 100 ml TAP medium [17] at 22.5°C under cool white fluorescent lights (4300 K) on a 14 L : 10 D cycle. We extracted DNA from dense cultures using the Qiagen^®^ DNeasy Plant Maxi Kit. Paired-end DNA libraries for Illumina sequencing were prepared by the Vincent J. Coates Genomics Sequencing Laboratory (Berkeley, CA, USA) using an Apollo 324™ Next Generation Sample Preparation System with the PrepX ILM 32i DNA Library Kit and sequenced on an Illumina^®^ HiSeq^®^ 2000.

Paired-end reads from Jang & Ehrenreich [16] (NCBI Short Read Archive accession numbers SRR516510-23 and SRR552312-837) and from our sequencing described in the previous paragraph were filtered using Trimmomatic v. 0.11.219 as follows: adapter sequences and low-quality bases (less than 3) were removed, reads trimmed when the average quality of a 4 bp sliding window dropped below 15, and trimmed reads less than 36 bp long discarded. Surviving reads were aligned to the *C. reinhardtii* reference genome (v. 5.5 from Phytozyme v. 11, phytozome.jgi.doe.gov) using the mem function of BWA [18].

### 2.2. Bulked segregant analysis

We used bulked segregant analysis [19] to identify alleles associated with multicellularity. We suspected that WRC01 was mating type (mt) minus, because no reads from the whole-genome sequencing aligned with the *FUS1* locus, which is diagnostic for mt+. Mating was carried out using the protocol in Harris [17], modified as follows: WRC01, CC-125 (mt+) and CC-124 (mt−) were grown separately in 50 ml TAP medium [20] shaken at 200 r.p.m. for 5 days. Cells were collected by centrifugation at 2000*g* for 5 min, resuspended in 25 ml *Chlamydomonas* mating medium [17], and grown under continuous light for 6 h to induce gametogenesis. Gametes were mixed in 6-well tissue culture plates as follows: each plate contained 4 wells with 2 ml each of WRC01 and either CC-125 or CC-124 and 2 wells with 4 ml of a single strain. The single-strain wells served as controls to ensure that no vegetative material survived (and thus that all surviving progeny were outcrossed). Plates were kept in continuous light for 24 h then in dark for 6 days, at which time all wells were completely desiccated. Zygotes were germinated by addition of 4 ml TAP medium per well and grown on a 14 L : 10 D cycle. Within three weeks, all four wells of the WRC01 × CC-125 cross showed obvious signs of growth, while none of the single-strain controls or the WRC01 × CC-124 wells showed any sign of growth, confirming that WRC01 is indeed mt−.

Pools of WRC01 × CC-125 F1 progeny were crossed to generate a large pool of F2 progeny for use in bulked segregant analysis. We separated F2 progeny into primarily unicellular and primarily multicellular pools by four rounds of growth and centrifugation. Ten millilitres of pooled algae were transferred to a 15 ml conical tube and centrifuged for 1 min at 100*g*. The top 2 ml (for the unicellular pool) or the bottom 1 ml (for the multicellular pool) was used to inoculate 50 ml TAP medium. We extracted total DNA from unicellular and multicellular pools using the DNeasy^®^ Plant Mini Kit (Qiagen). DNA libraries were generated by the Vincent J. Coates Genomics Sequencing Laboratory (Berkeley, CA, USA) using the PrepX ILM 32i DNA Library Kit and were sequenced on an Illumina HiSeq 2500 at the Georgia Institute of Technology. The resulting sequence data are available on the NCBI Sequence Read Archive as accession numbers SAMN09580655–6.

Adapter sequences and low-quality bases were removed, reads were trimmed, and trimmed reads less than 36 bp long were dropped using Trimmomatic v. 0.11.2 as described above for the whole-genome sequencing analysis [21]. Paired-end reads for which both reads survived filtering were aligned to the *C. reinhardtii* genome v. 5.5 (phytozyme.jgi.doe.gov) using BWA-mem v. 0.7.10 [18]. Reads that aligned with a mapping quality less than 30 were removed using SamTools v. 0.1.15 [22].

SNPs identified as differing between WRC01 and CC-125 were treated as alleles segregating in the F2 population. Alleles originating in WRC01 and overrepresented in the multicellular pool are candidates for causing the multicellular phenotype in WRC01, and we quantified the degree of their overrepresentation in terms of log odds. Odds and log odds for allele frequency differences between the two bulked segregant pools were calculated using exact binomial probabilities as follows: given the overall frequency of an allele across the two pools, what are the odds of observing the estimated frequency difference (or greater) between the two pools by chance? We chose to estimate deviations from the observed overall allele frequency, rather than deviations from the expected 1 : 1 frequency, in case some alleles provided a growth rate advantage and consequently increased in frequency while cells were being grown to high density for DNA extractions. Deviations from the observed overall frequency can only be due to sampling error or to selection, and the statistical test was designed to distinguish between these possibilities.

### 2.3. RNA-Seq

We extracted total RNA using the RNeasy^®^ Plant Mini Kit (Qiagen) from three biological replicates each of WRC01 and CC-125 at 3, 6, 9, 12 and 48 h post-inoculation. These time points were selected to compare gene expression in each phase of the multicellular clone's life cycle: lag (3 h), release of swimming propagules (6 and 9 h) and growth and development of multicellular clusters (12 and 48 h). Cultures for RNA extraction were grown under conditions identical to those in which the experiment was carried out. Cells were collected by centrifugation at 17 000*g* for 1 min, snap-frozen in liquid N_2_ and stored at −80°C for up to one week before extraction. Cells were not disrupted as specified in the RNeasy protocol, but rather were thawed and immediately mixed with lysis buffer by pipetting. RNA quality was quantified using an Agilent 2100 Bioanalyzer using the Plant RNA Nano assay.

We generated cDNA libraries from 500 ng total RNA using the TruSeq^®^ RNA Sample Preparation Kit v. 2 (Illumina^®^) following the standard protocol, with the following exceptions. cDNA fragments were enriched using 14 PCR cycles, based on the results of qPCR using the DyNAmo Flash SYBR Green qPCR Kit (Thermo Scientific). Clusters and 100 bp paired-end reads were generated on an Illumina Hi-Seq 2500 at the Georgia Institute of Technology. The RNA-Seq experiment was blocked by flow cell, such that each of the three Hi-Seq flow cells contained one replicate of each strain at each time point. Lane assignments and Illumina indices (from the TruSeq RNA Sample Preparation Kit) are shown in electronic supplementary material, table S1.

Adapter sequences and low-quality bases (less than 3) were removed, reads trimmed when the average quality of a 4 bp sliding window dropped below 15 and trimmed reads less than 36 bp long dropped using Trimmomatic v. 0.11.2 [21]. Differential gene expression was estimated using the Cufflinks workflow [23]. Paired-end reads for which both reads survived Trimmomatic filtering were mapped to the *Chlamydomonas* reference genome (Phytozome 5.5) using TopHat v. 2.0.13 [23,24], transcriptomes assembled using Cufflinks v. 2.2.1 [23], transcript expression quantified using Cuffquant v. 2.2.1 [23] and expression differences quantified using Cuffdiff v. 2.2.1 [23].

Expression levels, estimated as Fragments Per Kilobase of transcript per Million mapped reads (FPKMs), among the three biological replicates from each strain (CC-125 and WRC01) at each time point (3, 6, 9, 12 and 48 h), were correlated with *r* > 0.98 in all pairwise comparisons (electronic supplementary material, table S2). The raw RNA-Seq data are available from the NCBI Sequence Read Archive accession numbers SAMN09580649–50. For the following analyses, we included only differentially expressed genes with an average expression level difference of fourfold or greater (log_2_ ≥ 2) at one or more time points.

We estimated the phylogenetic origins (phylostrata [25]) of differentially expressed genes using protein–protein BLAST [26] searches of translated primary transcripts from the *C. reinhardtii* reference genome (Phytozome 5.5). BLASTp matches with an *E*-value < 0.001 were considered homologous. Homologues were assigned to one of the following categories (in order of increasing inclusiveness) according to the GenBank taxonomy: *Chlamydomonas*, volvocine, Chlamydomonadales, Chlorophyceae, Chlorophyta, Viridiplantae, Eukaryota and cellular organisms (figure 3). We tested variation from expected frequencies of differentially expressed genes from particular phylostrata using a two-tailed hypergeometric test with the Bonferroni correction (*α* = 0.0025, equivalent to a false discovery rate of 1%) in GeneMerge v. 1.4 [27]. Expected frequencies for this analysis were those assigned to a taxonomic level across the entire genome, to test the null hypothesis that differentially expressed genes of a particular taxonomic level are represented at their underlying (genome-wide) frequency.

Functional annotation of differentially expressed genes was carried out using Blast2GO v. 3.3.5 [28,29]. We tested variation from expected (genome-wide) frequencies of differentially expressed genes with particular ontologies using a two-tailed hypergeometric test (*α* = 0.0025, equivalent to a false discovery rate of 1%) in GeneMerge v. 1.4 [27].

## 3. Results

### 3.1. Whole-genome sequencing

To identify and characterize genetic variants, we sequenced the evolved multicellular strain and several parental strains. Paired-end Illumina sequencing generated an average depth of coverage of 47× across the WRC01 genome. Average coverage for the parental strains CC-124, CC-1690, CC-1691 and CC-4414 was 70×, 39×, 36× and 61×, respectively. The WRC01 genome contains large-scale differences from all of the parental strains used to found the starting populations, confirming that it descends from recombined sexual progeny.

Because the experimental populations were founded from a genetically diverse pool of outcrossed progeny, there is no single ancestral genotype to which we can compare the evolved multicellular strain. Genetic variants contributing to the multicellular phenotype could be novel mutations that occurred during the experiment, novel combinations of alleles that were present in the parental strains or a combination of both. Variants identified by whole-genome sequencing must therefore be tested for association with the multicellular phenotype.

### 3.2. Bulked segregant analysis identifies genomic regions associated with undifferentiated multicellularity

**Figure 1.**
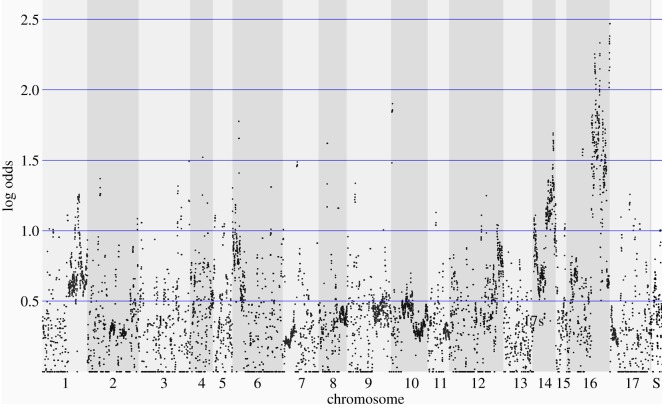
Results of bulked segregant analysis. Each point represents a non-synonymous SNP. Log odds reflect the probability of observing the estimated frequency difference (or greater) between the two pools by chance. ‘S’ refers to scaffolds not assembled into a chromosome.

To distinguish genetic variants contributing to the multicellular phenotype from those that are unrelated, we used bulked segregant analysis of an F2 population generated by crossing the multicellular strain WRC01 and unicellular CC-125. Average depth of coverage for the bulked segregant pools enriched for unicellular and multicellular algae were 155× and 128×, respectively. Results from the bulked segregant analysis are shown in figure 1. Markers on portions of chromosomes 14 and 16 are substantially overrepresented in the bulked segregant pool enriched for multicellular algae.

### 3.3. RNA-Seq analysis of unicellular and multicellular growth cycles reveals differential expression of lineage- and group-specific genes

**Figure 2.**
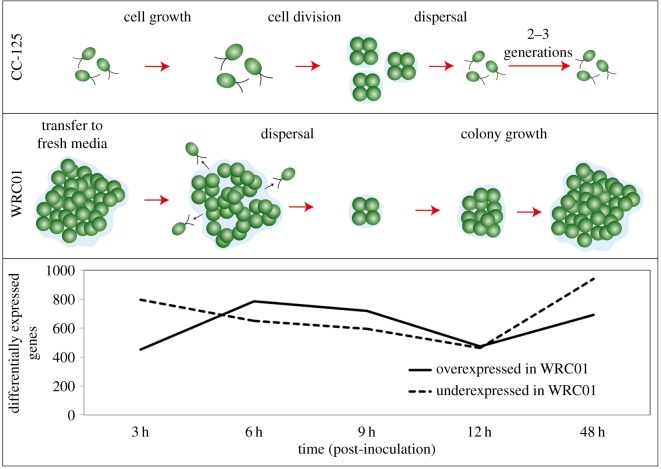
Number of genes (out of 17 741) greater than fourfold differentially expressed in multicellular *C. reinhardtii* (WRC01) compared to unicellular *C. reinhardtii* (CC-125) and approximate depictions of life cycle stage at each time point.

To gain insight into gene expression differences underlying de novo multicellularity, we estimated transcript levels in WCR01 and compared them to those of wild-type *C. reinhardtii* (CC-125). Previous experiments showed that the multicellular clone had evolved a characteristic life cycle, discernible over 72 h, including an approximately 4 h lag followed by release of unicellular propagules (approx. 4 to 6 h), which developed into multicellular clusters over the next approximately 48 h. To capture this process, we inoculated three biological replicates each of a unicellular ancestor (CC-125) and the multicellular clone (WCR01), harvested cells at 3, 6, 9, 12 and 48 h and subjected each to RNA-Seq analysis. FPKM values from the three biological replicates of each combination of strain and time point were highly correlated (*R*^2^ > 0.96 in every case). We found large-scale expression differences between the evolved multicellular isolate WRC01 and unicellular CC-125 (figure 2).

**Figure 3.**
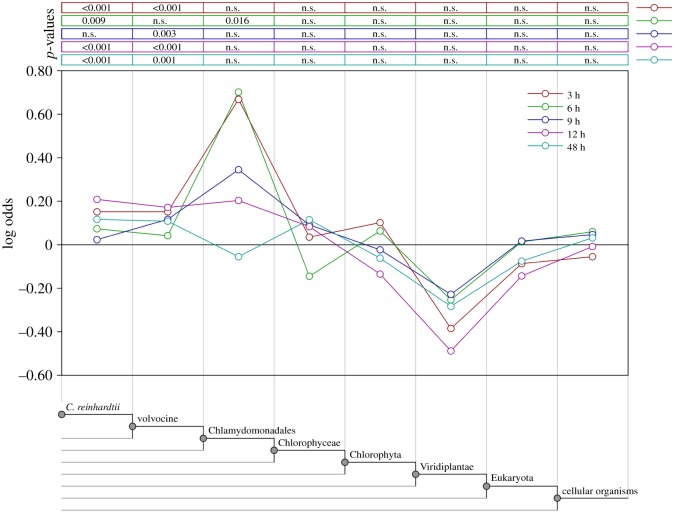
Results of phylostratigraphy analysis. The *y*-axis represents the log odds of the observed degree of over/underrepresentation relative to genome-wide frequencies. The Bonferroni-corrected *p*-values result from a hypergeometric test (*α* = 0.0025, equivalent to a false discovery rate of 1%) performed in GeneMerge v. 1.4 [27]. ‘n.s.’, not significant.

Genes whose transcript levels differ by at least fourfold between CC-125 and WRC01 are significantly enriched for volvocine-specific (*Chlamydomonas* *+* *Gonium* *+* *Volvox*) genes compared to the *C. reinhardtii* genome (figure 3). *Chlamydomonas reinhardtii*-specific genes were significantly overrepresented at 3, 6, 12 and 48 h, and volvocine-specific genes were significantly overrepresented at 3, 9, 12 and 48 h. Chlamydomonadales-specific genes appear to be overrepresented at the first four time points, but only significantly at 6 h. The Chlamydomonadales results are possibly due to sampling bias: the only published genomes in the Chlamydomonadales are in *Chlamydomonas* and volvocine species, so only 13 genes were assigned to this phylostratum genome-wide, when compared with greater than 6000 to *C. reinhardtii* and greater than 2000 to volvocine.

Among the significantly differentially expressed genes, 3453 were over/underexpressed by fourfold or greater at one or more time points. Table 1 shows gene ontology categories that are overrepresented among differentially expressed genes, relative to their representation in the *C. reinhardtii* genome. Of particular interest is the overrepresentation of gene ontologies related to cell cycle and reproductive processes downregulated at 3 h post-inoculation and upregulated at 9 h. Consistent with the observation that multicelled clusters undergo little or no cell division during the first 4 h post-inoculation, several gene ontologies related to mitosis are significantly overrepresented among genes underexpressed at 3 h. These include cell cycle (GO:0007049), cell division (GO:0051301), DNA replication (GO:0006260), nuclear division (GO:0000280), cell division (GO:0051301), cell proliferation (GO:0008283), regulation of cell division (GO:0051302) and regulation of cell cycle (GO:0051726). With the exception of regulation of cell division, the same ontologies are overrepresented among the genes overexpressed at 9 h, when clusters are beginning to grow through mitosis. In addition, some of the gene ontologies overrepresented among the upregulated genes at 6 and 9 h are associated with multicellular developmental processes, including regulation of post-embryonic development (GO:0048580), regulation of reproductive process (GO:2000241) and regulation of shoot system development (GO:0048831). A complete list of the gene ontologies making up each category and the complete results of the GeneMerge [27] analyses, including the differentially expressed genes in each ontology, are available on Dryad: http://dx.doi.org/10.5061/dryad.6447n78 [30].

**Table 1.** Gene ontology categories overrepresented among differentially expressed genes (greater than or equal to fourfold differential expression; false discovery rate ≤1%). Headings describe the time point and direction of differential expression, e.g. 3 h + 4x refers to genes overexpressed by at least fourfold at 3 h post-inoculation.

description	3 h + 4×	6 h + 4×	9 h + 4×	12 h + 4×	48 h + 4×	3 h−4×	6 h−4×	9 h−4×	12 h−4×	48 h−4×
cAMP metabolic process										
cell cycle										
cell cycle phase transition										
cell division										
cell proliferation										
cellular aromatic compound metabolic process										
cellular component organization or biogenesis										
cellular macromolecule localization										
cellular macromolecule metabolic process										
cellular metabolic process										
cellular process (excluding cellular metabolic process)										
cellular response to DNA damage stimulus										
cellular response to stress										
cGMP biosynthetic process										
cGMP metabolic process										
chromatin modification										
chromosome condensation										
chromosome organization										
chromosome segregation										
chromosome separation										
covalent chromatin modification										
cyclic purine nucleotide metabolic process										
cytoskeleton organization										
DNA catabolic process, endonucleolytic										
DNA metabolic process										
DNA modification										
DNA recombination										
DNA replication										
DNA replication, synthesis of RNA primer										
DNA strand elongation										
establishment of protein localization										
gene silencing										
GTP metabolic process										
heat acclimation										
heterocycle metabolic process										
inorganic anion transmembrane transport										
maturation of 5.8S rRNA										
meiotic cell cycle										
meiotic chromosome segregation										
methylation										
microtubule-based process										
mitochondrial transport										
mitotic cell cycle										
negative regulation of metabolic process										
nitrogen compound metabolic process										
nuclear division										
nuclear transport										
nucleobase-containing compound transport										
organelle organization										
organic cyclic compound metabolic process										
organic substance metabolic process										
peptidyl-amino acid modification										
phosphate ion transmembrane transport										
plastid fission										
primary metabolic process										
protein localization to nucleus										
recombinational repair										
regulation of cell cycle										
regulation of cell division										
regulation of DNA metabolic process										
regulation of post-embryonic development										
regulation of reproductive process										
regulation of shoot system development										
reproduction										
ribonucleoprotein complex biogenesis										
ribonucleoprotein complex localization										
RNA localization										
single-organism cellular localization										
single-organism organelle organization										
somatic cell DNA recombination										
spindle organization										

## 4. Discussion

The life cycle of the multicellular isolates described in Ratcliff *et al.* [12] alternates between unicellular and multicellular stages. The time points used in the RNA-Seq experiment were chosen to bracket milestones in the life cycle: at 3 h, clusters appear dormant; at 6 h, unicellular propagules are actively swimming; at 9 and 12 h, many have lost motility and begun to develop into multicellular clusters; and at 48 h, clusters have reached a large size (approx. 50 cells or more).

Many of the gene ontologies related to mitosis that are overrepresented among genes underexpressed at 3 h are also overrepresented among genes overexpressed at 9 h, including several related to cell cycle and reproductive processes. This is consistent with observations of the evolved multicellular life cycle, in which cell division is largely absent at 3 h but rampant at 9 h. The overrepresentation of gene ontologies related to multicellular development at 6 and 9 h is also consistent with the observed initiation of multicellular clusters at around this time.

Among differentially expressed genes, either volvocine-specific or Chlamydomonadales-specific genes are overrepresented at all five time points, suggesting that the genetics underlying the evolution of multicellularity in our experiment [12] has more in common with that in *Volvox* and related algae than with land plants. By contrast, green algal (Chlorphyceae/Chlorophyta) and Viridiplantae-specific genes are not overrepresented at any time point. In spite of a very different resulting phenotype, *C. reinhardtii* selected for multicellularity nevertheless followed a genetic pathway similar to that of the multicellular volvocine algae. However, *C. reinhardtii*-specific genes are also overrepresented at four of the five time points, suggesting important differences from volvocine evolution.

The results of the bulked segregant analysis show that at least two loci, on chromosomes 14 and 16, are significantly associated with the multicellular phenotype. While this analysis did not have sufficient resolution to identify the specific mutations responsible for the phenotype, this analysis considerably narrows the range of possibilities.

Understanding the evolution of complexity requires exploring both the ultimate (selective) and proximate (mechanistic) causes. The experiments described here, along with previous experiments by ourselves [11,12] and others [9,31,32], have shown that single-celled organisms can readily evolve multicellularity when subjected to the right selective pressures. We have shown that the genetic basis for the experimental evolution of *C. reinhardtii* involves large-scale changes in gene expression affecting nearly 20% of the organism's transcriptome. Conspicuous among these transcripts are those encoded by genes involved in multicellular development. The observed genetic changes have more in common with multicellular volvocine algae than with other multicellular green algae or land plants, but a large portion were *Chlamydomonas*-specific genes as well.

## Supplementary Material

Supplemental Table 1

## Data accessibility

Nucleotide sequence data are archived at the National Center for Biotechnology Information Sequence Read Archive under accession numbers SAMN09580649–56. Gene expression data, variants detected in the bulked segregant analysis, code and full results of the phylostratigraphy analysis and gene functional annotation are archived at Dryad: http://dx.doi.org/10.5061/dryad.6447n78 [30].
