# Supplementary material for: Genetics of a de novo origin of undifferentiated multicellularity
Source: R Soc Open Sci. 2018 Aug 29;5(8):180912. doi: 10.1098/rsos.180912 (PMC6124120; doi:10.1098/rsos.180912)
Supplement: Supplemental Table 1 [file rsos180912supp1.docx]

Table S1. Illumina indices of RNA-Seq samples.

| Strain | Time point (h) | Index | Lane |
| --- | --- | --- | --- |
| CC-125 | 3 | 4 | 1 |
| CC-125 | 6 | 14 | 1 |
| CC-125 | 9 | 2 | 1 |
| CC-125 | 12 | 15 | 1 |
| CC-125 | 48 | 5 | 1 |
| WRC01 | 3 | 7 | 1 |
| WRC01 | 6 | 18 | 1 |
| WRC01 | 9 | 6 | 1 |
| WRC01 | 12 | 19 | 1 |
| WRC01 | 48 | 12 | 1 |
| CC-125 | 3 | 5 | 2 |
| CC-125 | 6 | 15 | 2 |
| CC-125 | 9 | 4 | 2 |
| CC-125 | 12 | 16 | 2 |
| CC-125 | 48 | 6 | 2 |
| WRC01 | 3 | 12 | 2 |
| WRC01 | 6 | 19 | 2 |
| WRC01 | 9 | 7 | 2 |
| WRC01 | 12 | 2 | 2 |
| WRC01 | 48 | 13 | 2 |
| CC-125 | 3 | 6 | 3 |
| CC-125 | 6 | 16 | 3 |
| CC-125 | 9 | 5 | 3 |
| CC-125 | 12 | 18 | 3 |
| CC-125 | 48 | 7 | 3 |
| WRC01 | 3 | 13 | 3 |
| WRC01 | 6 | 2 | 3 |
| WRC01 | 9 | 12 | 3 |
| WRC01 | 12 | 4 | 3 |
| WRC01 | 48 | 14 | 3 |

Table S2. Correlations (*r*) between FPKMs of biological replicates.

| Time | CC-125  1 *v* 2 | CC-125  2 *v* 3 | CC-125  1 *v* 3 | WRC01  1 *v* 2 | WRC01  2 *v* 3 | WRC01  1 *v* 3 |
| --- | --- | --- | --- | --- | --- | --- |
| 3h | 0.999 | 0.994 | 0.997 | 0.999 | 0.987 | 0.987 |
| 6h | 0.999 | 0.996 | 0.997 | 0.998 | 0.984 | 0.986 |
| 9h | 0.995 | 0.999 | 0.999 | 0.999 | 0.999 | 0.999 |
| 12h | 0.999 | 0.999 | 0.996 | 0.998 | 1.000 | 0.998 |
| 48h | 0.992 | 0.997 | 0.983 | 0.999 | 0.998 | 0.999 |
